# Radiological Diagnosis of a Rare Premature Aging Genetic Disorder: Progeria (Hutchinson-Gilford Syndrome)

**DOI:** 10.1155/2017/1305360

**Published:** 2017-09-12

**Authors:** Haji Mohammed Nazir, Akshiitha Ramesh Baabhu, Yuvaraj Muralidharan, Seena Cheppala Rajan

**Affiliations:** Saveetha Medical College and Hospital, Saveetha Nagar, Thandalam, Chennai, Tamil Nadu 602105, India

## Abstract

Hutchinson-Gilford Progeria Syndrome (HGPS) is a rare disease with a combination of short stature, bone abnormalities, premature ageing, and skin changes. Though the physical appearance of these patients is characteristic, there is little emphasis on the characteristic radiological features. In this paper, we report a 16-year-old boy with clinical and radiological features of this rare genetic disorder. He had a characteristic facial appearance with a large head, large eyes, thin nose with beaked tip, small chin, protruding ears, prominent scalp veins, and absence of hair.

## 1. Introduction

Hutchinson-Gilford Progeria Syndrome is an extremely rare genetic disease, which was named after the two doctors who first described it in England, in 1886 by Dr. Hutchinson and in 1897 by Dr. Hastings Gilford [1]. The reported prevalence rate of progeria is one in eight million births and if unreported cases are taken into account, the estimated birth prevalence is one in four million [2]. According to the Progeria Research Foundation database as of January 2017, there are 144 progeric children worldwide, residing in 46 countries [3]. The average survival age is 13.5 years (life expectancy about 8–21 years) and death occurs mostly due to stroke, myocardial infarction, heart failure, or atherosclerosis [4].

## 2. Case Presentation

A 16-year-old boy, born to nonconsanguineous parents presented to our hospital with difficulty in walking and bilateral hip deformity, following a trivial trauma, four months before the presentation. The boy was short and had a bird-like facial appearance (Figure 6). History from the mother revealed that perinatal period was uneventful and had two siblings who were normal. On general examination, the boy had prominent eyes, prominent scalp veins, small chin, small face and large head, beaked nose, loss of subcutaneous fat, muscular atrophy, kyphosis, brown pigmentation all over the trunk, coarse thickened skin, broad stumpy fingers, and short wide toes. The boy resembled a little old person with loss of scalp hair, eyebrows, and eyelashes. He had normal intelligence. The first visible abnormality his parents noticed was excessive hair loss at three months of age. Based on the history and clinical diagnosis, a provisional diagnosis of progeria was made.

**Figure 1:**
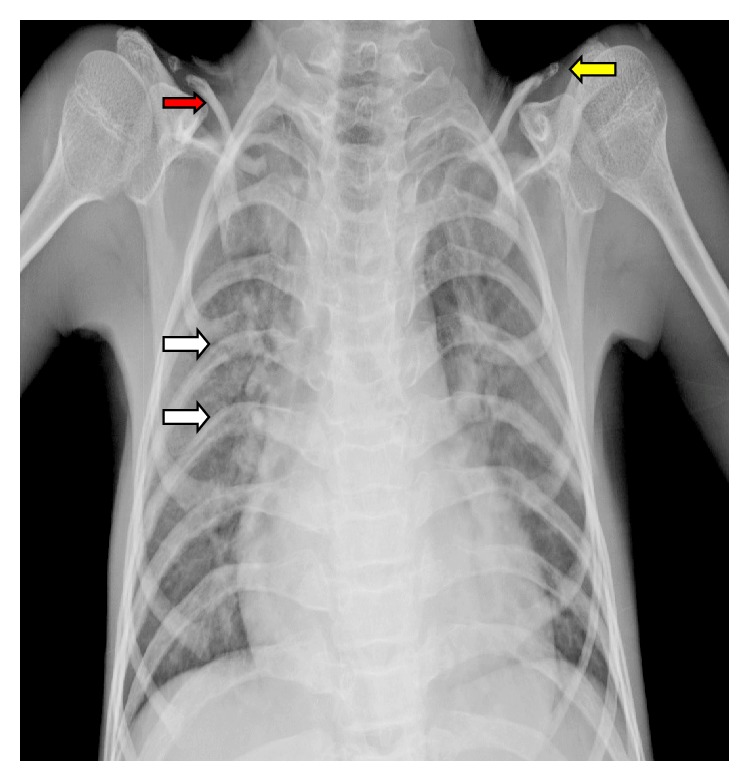
Frontal chest radiograph showing cardiomegaly, sloping slender fifth and sixth posterior ribs (white arrows). The absence of lateral and medial end of both clavicles (red arrow) with a tiny bone fragment was seen in the acromial end of both clavicles (yellow arrow).

**Figure 2:**
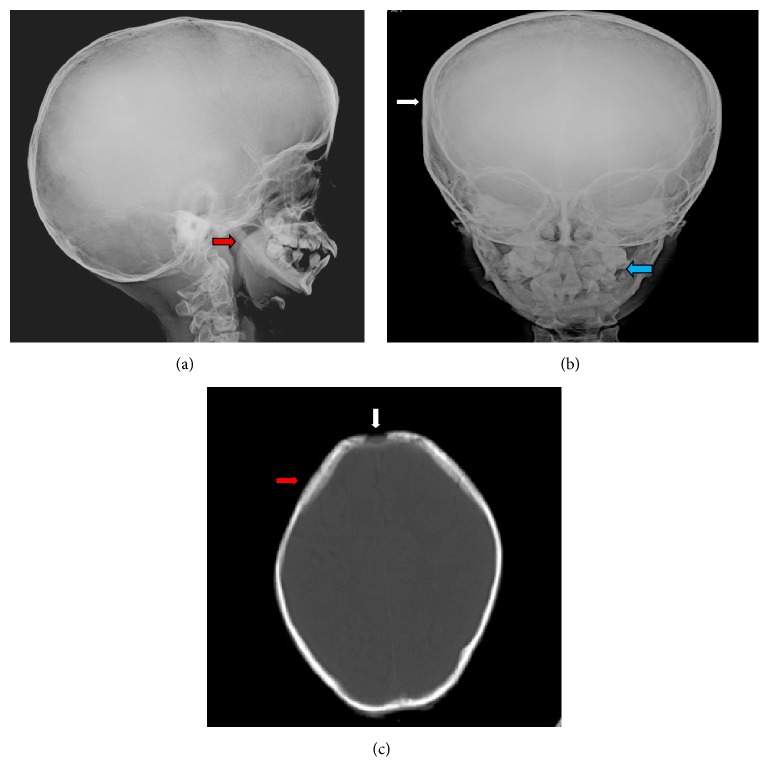
Skull radiograph lateral and anteroposterior views (a, b) showing disproportionate large calvarium with hypoplastic facial bones, small mandible with small ascending ramus, and obtuse mandibular angle (red arrow) and overcrowding of teeth (blue arrow). CT head (c) showing open anterior fontanelle (white arrow) and large cranial vault with thin diploic space (red arrow).

**Figure 3:**
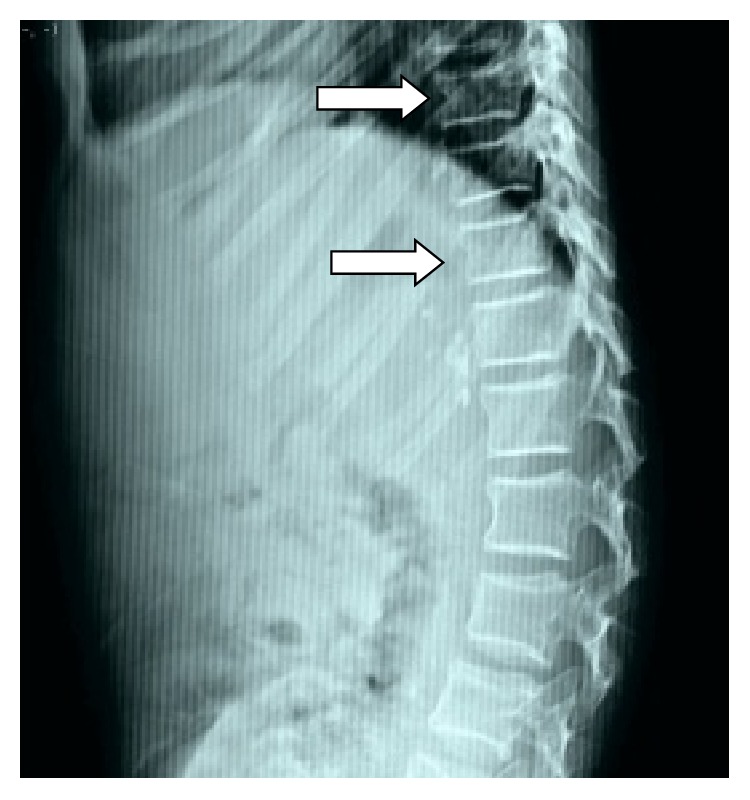
Radiograph of the dorsal spine (lateral projection) showing thoracic kyphosis and mild concavity in the anterior aspect of vertebral bodies (arrows). Prevertebral calcific foci seen adjacent to lower thoracic vertebrae.

**Figure 4:**
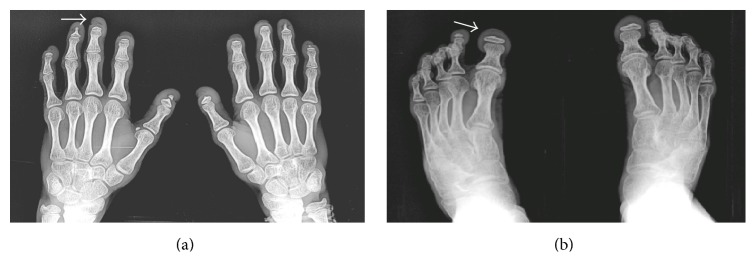
Anteroposterior radiograph of both hands (a) and foot (b) showed resorption of terminal phalanges with preservation of soft tissue (white arrow).

**Figure 5:**
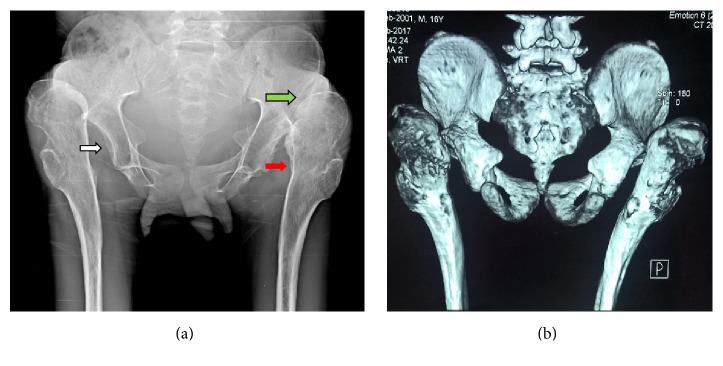
Anteroposterior radiograph of pelvis with both hip joints (a) showed widening of pubic symphysis, bilateral shallow acetabulum (white arrow), abnormal contour of bilateral femoral head with increased neck shaft angle (coxa valga deformity) (red arrow), enlarged and elongated bilateral greater trochanter, superolateral migration of bilateral femoral head overlying adjacent to iliac bones (green arrow), and slender proximal femoral shafts. CT of pelvis (b) confirming pubic diastasis, bilateral shallow acetabulum, bilateral coxa valga deformity, bilateral hip dislocation, and pseudoarticulation of the left femoral head with iliac bone.

**Figure 6:**
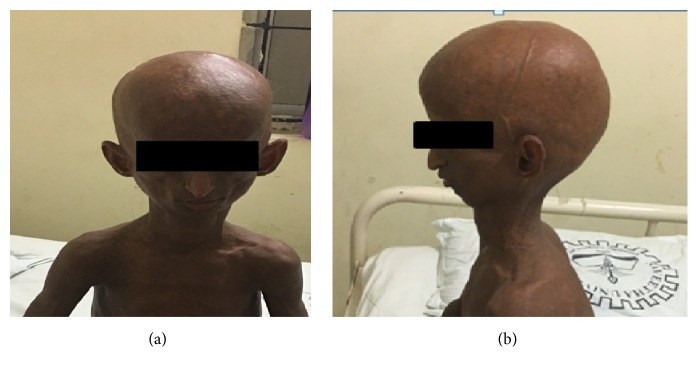
Real life pictures of the 16-year-old male patient with bird-like facies: (a) en face view and (b) profile view.

Routine biochemical tests were normal except for elevated serum cholesterol. The patient was sent to our radiology department and was subjected for the skeletal survey. Frontal chest radiograph showed cardiomegaly, sloping slender fifth, and sixth posterior ribs. The absence of lateral and medial ends of both clavicles with a tiny bone fragment was seen in the acromial ends of both clavicles (Figure 1). Skull radiograph in lateral view showed disproportionate large calvarium with hypoplastic facial bones, small mandible with small ascending ramus, and obtuse mandibular angle with the overcrowding of teeth (Figures 2(a) and 2(b)). CT head showed open anterior fontanelle, large cranial vault with thin diploic space, and no abnormality in the brain (Figure 2(c)). Radiograph of the dorsal spine in the lateral projection showed the presence of mild concavity in the anterior aspect of vertebral bodies (“fish mouth” vertebra) and kyphotic curvature [5]. Prevertebral calcifications are shown in Figure 3. Radiograph of both hands' anteroposterior view showed resorption of terminal phalanges with preservation of soft tissue (Figure 4(a)). Radiograph of both feet's anteroposterior view showed resorption of terminal phalanges with preservation of soft tissue (Figure 4(b)). Radiograph of pelvis with both hip joints in anteroposterior view showed widening of pubic symphysis, bilateral shallow acetabulum, abnormal contour of bilateral femoral head with increased neck shaft angle (coxa valga deformity), enlarged and elongated bilateral greater trochanter, superolateral migration of bilateral femoral head overlying adjacent to iliac bones, and slender proximal femoral shafts (Figure 5(a)). Further imaging with computed tomography (CT) of pelvis confirmed the radiographic findings of pubic diastasis, bilateral shallow acetabulum, bilateral coxa valga deformity, bilateral hip dislocation, and pseudoarticulation of the left femoral head with iliac bone (Figure 5(b)). Bones appeared diffusely osteopenic in all the radiographs.

## 3. Discussion

Progeria is a genetic disease which is extremely rare and characterised by accelerated premature ageing. Gilford in 1904 coined the term progeria which is derived from the Greek word “geros” meaning old. In progeria the pattern of premature aging is segmental as cataracts, Alzheimer's disease or cancers typical of aging do not occur in progeric children. Also the typical balding pattern and bone changes of progeria do not occur with normal aging [6]. The affected individual ages seven times faster than that of normal individuals. The disease occurs due to the sporadic mutation in the LMNA gene, which provides instructions for making proteins lamins A and C. These proteins are the main components of intermediate filamentous lamina which function as a structural support [7]. This disorder is confirmed by a genetic test for the point mutation in LMNA gene [8]. Genetic analysis was not performed for this case due to lack of such facilities in and around our institution. Our case was mainly diagnosed by classical clinical and characteristic radiology features. Elevated levels of serum triglycerides, total cholesterol low-density lipoprotein cholesterol, and reduced levels of high-density lipoprotein cholesterol have been reported [9]. Elevated levels of hyaluronic acid excretion in the urine may be seen but are not diagnostic.

Progeric children are normal at birth and within the first year of life develop severe growth deficiency with sclerodermatous skin changes. Loss of subcutaneous fat with alopecia changes in the skin give the typical “plucked-bird” appearance at about 6–12 months of age. Simultaneously, scalp hair and eyelashes are progressively lost and finally resulting in baldness with prominent scalp veins. Abnormal dentition, beaked nose, receding chin, and exophthalmos are the typical facial features in these patients. Muscular atrophy with joint deformities is additional features. Intelligence is normal or above normal in these patients. All these physical features were seen in our patient, giving a physical appearance of “a wizened little old man.” Commonest cause of death in progeria is heart disease due to genetic predisposition in the affected persons.

A review of the previously reported radiography findings in affected individuals with progeria disclosed a notable similarity of changes [10]. Diffuse osteopenia, prominent vascular markings in skull, thin and large calvarium with shallow diploic space, multiple wormian bones, small mandible with infantile obtuse angle and short ascending rami, hypoplastic facial bones, open cranial fontanelles, thin short clavicles, dwarfism, abnormally gracile ribs involving the posterior segments of the upper ribs, slender long bones, kyphosis, coxa valga, and progressive acroosteolysis of the terminal phalanges were the major roentgen findings previously reported. All the above findings were seen in our patient, except wormian bones. Bilateral coxa valga deformity with bizarre greater trochanters and shallow acetabulum with bilateral hip dislocation were seen in our patient. Coxa magna, coxa valga, acetabular dysplasia, hip subluxation, and dislocation are the common orthopaedic hip manifestations which have been previously reported [11]. Avascular necrosis of the femoral head, flared long bone metaphysis/epiphysis, enlarged capitulum of the distal humerus have also been reported.

The radiological findings of our case were consistent with those of which were present in more than fifty percent of cases of a largely appreciated study by Cleveland et al. on HGPS [12].

This discussion will be incomplete without a mention on “nonclassical” progeria in which the patients show less growth retardation, presence of scalp hair for a longer time, more slowly progressive lipodystrophy, and survival well into adulthood [13].

Differential diagnoses are other ageing syndromes like Werner's syndrome (adult progeria), Cockyane's syndrome, Acrogeria, and Rothmund-Thomson syndrome which can be differentiated by their clinical features. Progeric individuals rarely survive beyond the second decade and the cause of death is usually due to myocardial infarction or congestive heart failure.

## 4. Conclusion

The presented case provides important teaching points with regard to clinical and radiological findings of progeria syndrome which is an extremely rare genetic disorder with fatal outcome. Adequate knowledge on the radiological findings can aid in excluding mimics of progeria at diagnosis. The rarity of the disease and limited availability of literature prompted us to report this case.

## References

[1] Hutchinson J. (1886). Case of congenital absence of hair, with atrophic condition of the skin and its appendages, in a boy whose mother had been almost wholly bald from alopecia areata from the age of six. *Lancet*.

[2] Pollex R. L., Hegele R. A. (2004). Hutchinson-Gilford progeria syndrome. *Clinical Genetics*.

[3] Progeria Research Foundation (PRF) webpage, http://www.progeriaresearch.org/assets/files/pdf/PRF-By-the-Numbers_-Jan-2017-Update.pdf

[4] Pereira S., Bourgeois P., Navarro C. (2008). HGPS and related premature aging disorders: From genomic identification to the first therapeutic approaches. *Mechanisms of Ageing and Development*.

[5] Rastogi R., Chander Mohan S. M. (2008). Progeria syndrome: a case report. *Indian Journal of Orthopaedics*.

[6] Progeria Research Foundation (PRF) webpage, https://www.progeriaresearch.org/assets/files/pdf/info_sheets/18_ProgeriaAging_0410.pdf

[7] Shumaker D. K., Kuczmarski E. R., Goldman R. D. (2003). The nucleoskeleton: lamins and actin are major players in essential nuclear functions. *Current Opinion in Cell Biology*.

[8] Eriksson M., Brown W. T., Gordon L. B. (2003). Recurrent de novo point mutations in lamin A cause Hutchinson-Gilford progeria syndrome. *Nature*.

[9] Sarkar P. K., Shinton R. A. (2001). Hutchinson-Guilford progeria syndrome. *Postgraduate Medical Journal*.

[10] Ullrich N. J., Silvera V. M., Campbell S. E., Gordon L. B. (2012). Craniofacial abnormalities in Hutchinson-Gilford Progeria syndrome. *American Journal of Neuroradiology*.

[11] Akhbari P., Jha S., James K. D., Hinves B. L., Buchanan J. A. (2012). Hip pathology in Hutchinson–Gilford progeria syndrome. *Journal of Pediatric Orthopaedics B*.

[12] Cleveland R. H., Gordon L. B., Kleinman M. E. (2012). A prospective study of radiographic manifestations in Hutchinson-Gilford progeria syndrome. *Pediatric Radiology*.

[13] Hennekam R. C. M. (2006). Hutchinson-Gilford progeria syndrome: review of the phenotype. *American Journal of Medical Genetics*.

